# Microbiota Diversification and Crash Induced by Dietary Oxalate in the Mammalian Herbivore *Neotoma albigula*

**DOI:** 10.1128/mSphere.00428-17

**Published:** 2017-10-18

**Authors:** Aaron W. Miller, Colin Dale, M. Denise Dearing

**Affiliations:** a Departments of Urology and Immunology, Cleveland Clinic, Cleveland, Ohio, USA; b Department of Biology, University of Utah, Salt Lake City, Utah, USA; University of Wisconsin—Madison

**Keywords:** oxalate, urinary stone disease, woodrat

## Abstract

Oxalate, broadly found in both dietary and endogenous sources, is a primary constituent in 80% of kidney stones, an affliction that has tripled in prevalence over the last 40 years. Oxalate-degrading bacteria within the gut microbiota can mitigate the effects of oxalate and are negatively correlated with kidney stone formation, but bacteriotherapies involving oxalate-degrading bacteria have met with mixed results. To inform the development of more effective and consistent bacteriotherapies, we sought to quantify the interactions and limits between oxalate and an oxalate-adapted microbiota from the wild mammalian herbivore *Neotoma albigula* (woodrat), which consumes a high-oxalate diet in the wild. We tracked the microbiota over a variable-oxalate diet ranging from 0.2% to 12%, with the upper limit approximating 10× the level of human consumption. The *N. albigula* microbiota was capable of degrading ~100% of dietary oxalate regardless of the amount consumed. However, the microbiota exhibited significant changes in diversity dynamically at the operational taxonomic unit (OTU), family, and community levels in accordance with oxalate input. Furthermore, a cohesive microbial network was stimulated by the consumption of oxalate and exhibited some resistance to the effects of prolonged exposure. This study demonstrates that the oxalate-adapted microbiota of *N. albigula* exhibits a very high level of degradation and tolerance for oxalate.

**IMPORTANCE** The bacteria associated with mammalian hosts exhibit extensive interactions with overall host physiology and contribute significantly to the health of the host. Bacteria are vital to the mitigation of the toxic effects of oxalate specifically as mammals do not possess the enzymes to degrade this compound, which is present in the majority of kidney stones. Contrary to the body of literature on a few oxalate-degrading specialists, our work illustrates that oxalate stimulates a broad but cohesive microbial network in a dose-dependent manner. The unique characteristics of the *N. albigula* microbiota make it an excellent source for the development of bacteriotherapies to inhibit kidney stone formation. Furthermore, this work successfully demonstrates methods to identify microbial networks responsive to specific toxins, their limits, and important elements such as microbial network cohesivity and architecture. These are necessary steps in the development of targeted bacteriotherapies.

## Abstract (precis)

The bacteria associated with mammalian hosts exhibit extensive interactions with overall host physiology and contribute significantly to the health of the host. Bacteria are vital to the mitigation of the toxic effects of oxalate specifically as mammals do not possess the enzymes to degrade this compound, which is present in the majority of kidney stones. Contrary to the body of literature on a few oxalate-degrading specialists, our work illustrates that oxalate stimulates a broad but cohesive microbial network in a dose-dependent manner. The unique characteristics of the *N. albigula* microbiota make it an excellent source for the development of bacteriotherapies to inhibit kidney stone formation. Furthermore, this work successfully demonstrates methods to identify microbial networks responsive to specific toxins, their limits, and important elements such as microbial network cohesivity and architecture. These are necessary steps in the development of targeted bacteriotherapies.

## INTRODUCTION

The mammalian gut is home to trillions of bacteria comprised of hundreds to thousands of interacting bacterial operational taxonomic units (OTUs) with myriad functions (1, 2). So extensive are the host-microbe interactions, that the microbiota is vital for the development of organs, the immune system, and for host metabolism (3, 4). As such, the gut microbiota plays a key role as a buffer to the negative effects of many dietary toxins (5–8). However, the extent of the toxin-buffering capacity and the effects of toxins on the whole microbiota are unknown. It is important to understand the interactions between dietary toxins and the gut microbiota for the development of targeted bacteriotherapies tailored to individual patients. Thus, to understand specific interactions between dietary toxins and the gut microbiota, it is necessary to focus on a sufficiently simplified system.

Oxalate is a simple organic acid that is broadly distributed among plants, is regularly consumed by mammalian herbivores and humans, and contributes to 80% of kidney stones (9–12). Despite the toxicity of oxalate to mammals, it cannot be degraded by mammalian enzymes (13). However, it can be metabolized by many gut bacteria, such as *Oxalobacter formigenes* and *Lactobacillus acidophilus*, among others (5, 14). For *O. formigenes* specifically, oxalate is a carbon and energy source for growth (5). The microbial degradation of oxalate in the mammalian gut primarily proceeds via a simple, two-step enzymatic reaction involving the enzymes oxalyl-coenzyme A (CoA) decarboxylase and formyl-CoA transferase to produce one molecule each of CO_2_ and formate (15, 16). Oxalate can also be toxic to some bacteria, inhibiting proliferation, and introduces a potential source of dynamic interactions within a complex gut microbiota (17, 18). Furthermore, powdered sodium oxalate can be added to a diet, allowing for the effects of a single dietary variable to be examined in animal studies (19, 20). Thus, the metabolism of dietary oxalate by the gut microbiota is a relatively simple process that excludes contributions from the host, introduces a potential source of dynamic interactions, and can be studied in isolation. However, mammals do produce oxalate as a terminal metabolite in the liver from certain dietary precursors, which can make its way into the gut, and must be accounted for in studies concerning the interaction between dietary oxalate and the gut microbiota (21, 22).

The mammalian herbivore *Neotoma albigula* (woodrat) naturally consumes a high 1.5% oxalate diet in the wild (8). This species harbors consortia of oxalate-degrading bacteria that are stimulated by the introduction of oxalate to the diet and can degrade large amounts of dietary oxalate even when provided at 9% of the diet by dry weight (19, 23, 24). This oxalate-degrading capacity is largely unique to *N. albigula* and thus makes for an excellent study system to examine the interactions between oxalate and the gut microbiota and inform the development of bacteriotherapy strategies to minimize renal oxalate exposure (23, 24).

The present study was designed to test the hypothesis that the gut microbiota of *N. albigula* harbors a distinct and cohesive microbial network involved in oxalate metabolism with a limited and quantifiable buffering capacity for the negative effects associated with oxalate exposure. Our objectives were to quantify the maximum tolerable dose of oxalate for *N. albigula*, quantify changes to gut microbiota diversity metrics across different dietary oxalate concentrations, and identify OTUs that exhibit a significant change in relative abundance associated with oxalate consumption.

## RESULTS

### Effect of oxalate on host physiology.

**FIG 1:**
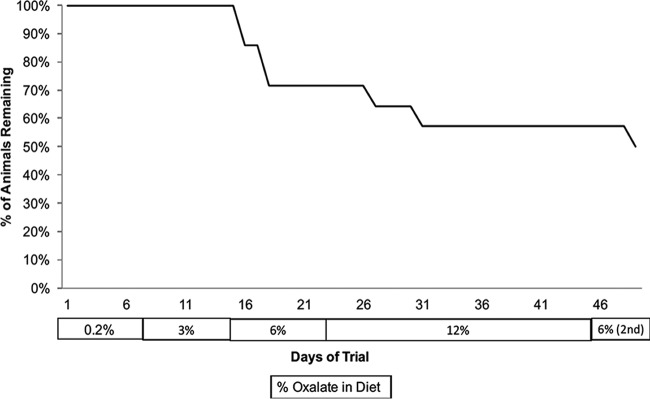
Kaplan-Meier survivor curve based upon the loss of 10% of body mass by *Neotoma albigula* on different oxalate diets.

**FIG 2:**
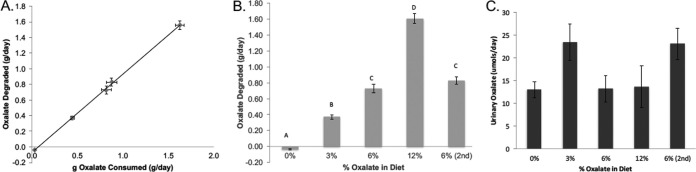
Oxalate handling for *N. albigula in vivo* for animals that completed the diet trial (*n =* 7). (A) The amount of oxalate consumed correlated with the amount of oxalate degraded (estimated by the differential between oxalate consumed and the total oxalate excreted in the urine and feces). Significance was determined as a repeated-measure Pearson correlation: *r* = 0.9993, *P* < 0.001. (B) Oxalate degradation across the diet trial. Data were analyzed with a repeated-measure ANOVA (df = 6.28, *P* < 0.001) and a *post hoc* Tukey’s analysis. Letters reflect statistical groups. (C) Daily urinary oxalate excretion for each dietary oxalate treatment. Data for the whole experiment were analyzed with a repeated-measure ANOVA (df = 6.28, *P* = 0.59).

A total of nine females and five males were used for the study (starting body masses of 171.15 ± 6.67 g and 195.22 ± 7.35 g, respectively). Seven out of the original 14 animals were removed from the trial starting after the first day of the 12% oxalate period, having lost >10% of their starting body mass, including three males and four females (Fig. 1). Animals remaining in the trial had a body mass change ranging from −4.3% to +8.8%. Those animals that persisted throughout the trial maintained ~100% oxalate degradation with no significant increase in urinary oxalate across all oxalate treatments in the experiment (Fig. 2). Of the woodrats that were removed from the diet trial, five out of seven exhibited an anomalously low level of oxalate degradation or high level of urinary oxalate at least once during the trial prior to dropping out. For woodrats that completed the diet trial, only two of the seven exhibited at least one anomalous value for oxalate degradation or urinary oxalate (Table 1). For oxalate degradation, anomalous values ranged from 1.3- to 2.7-fold lower than the average. For urinary oxalate, anomalous values were 1.8- to 2.5-fold higher than the average. However, differences in anomalous values between animals that were removed and those that completed the trial were not significant. Food intake, dry matter digestibility (DMD), fecal output, and fecal oxalate did not differ significantly over the course of the experiment. Dry matter digestibility is defined as (dry matter in the feed − dry matter in feces)/dry matter in the feed. Because there was no difference in these metrics over time, a global average is presented (see Table S1 in the supplemental material). Water intake and urine output did increase significantly with oxalate consumption (see Fig. S1 in the supplemental material).

10.1128/mSphere.00428-17.5 TABLE S1  Statistics for nonsignificant metrics. Global means are reported pooling all animals across all time points ± standard error. Data for each group and time point across the whole experiment were analyzed with a repeated-measure ANOVA. Download TABLE S1, PDF file, 0.1 MB. Copyright © 2017 Miller et al. 2017 Miller et al. This content is distributed under the terms of the Creative Commons Attribution 4.0 International license.

10.1128/mSphere.00428-17.1 FIG S1  Water intake and urine output for each dietary oxalate treatment. Data for the whole experiment were analyzed with a repeated-measure ANOVA. Letters indicate statistical groupings determined by a *post hoc* Tukey’s analysis. (A) Water intake (df = 6.28, *P* < 0.001); (B) urine output (df = 6.28, *P* < 0.001). Download FIG S1, PDF file, 0.1 MB. Copyright © 2017 Miller et al. 2017 Miller et al. This content is distributed under the terms of the Creative Commons Attribution 4.0 International license.

**TABLE 1:** Animals that exhibited an anomalously low value for oxalate degradation or high value for urinary oxalate excretion at some point in the diet trial and whether the animal was later removed^a^

Animal	Outlier diet (% oxalate)	Metric	Removal diet (% oxalate)
NALB 7	0	Urine oxalate	Not removed
NALB 10	0	Urine oxalate	12
NALB 8	3	Oxalate degradation	12
NALB 8	3	Urine oxalate	12
NALB 9	6	Urine oxalate	12
NALB 10	6	Oxalate degradation	12
NALB 12	6	Oxalate degradation	12
NALB 6	12	Oxalate degradation	Not removed
NALB 7	12	Urine oxalate	Not removed
NALB 13	12	Oxalate degradation	12
NALB 6	6 (2nd)	Urine oxalate	Not removed

a The diet based on percentage of oxalate on which the outlier occurred and the diet on which the animal was removed (if applicable) are indicated. The proportions of outliers between each group were compared with a Fisher’s exact test (*P* = 0.184).

### Effect of oxalate on gut microbiota.

Sequencing of fecal communities yielded 2,935,802 sequences after quality control. Across all samples, a total of 26,628 unique OTUs were defined. One animal was removed from the group that completed the trial as it did not produce a fecal sample for one of the time points. This resulted in a sample size of six animals with microbial inventories at each time point. Across OTUs, 93.4% were assignable to the phylum level, with 15.7% assignable to the genus level. The community was dominated by *Bacteroides*, particularly the S24-7 family (see Fig. S3 in the supplemental material).

10.1128/mSphere.00428-17.2 FIG S2  The relative abundance of *Oxalobacteraceae*. Relative abundance was evaluated with a repeated-measure ANOVA (5, 24) (*F* = 4.9198, *P* = 0.006). Download FIG S2, PDF file, 0.1 MB. Copyright © 2017 Miller et al. 2017 Miller et al. This content is distributed under the terms of the Creative Commons Attribution 4.0 International license.

10.1128/mSphere.00428-17.3 FIG S3  Phylum-level profile of the fecal microbiota over the duration of the experiment, for each animal that completed the trial. (A) NALB 1; (B) NALB 2; (C) NALB 3; (D) NALB 4; (E) NALB 5; (F) NALB 6. Download FIG S3, PDF file, 0.1 MB. Copyright © 2017 Miller et al. 2017 Miller et al. This content is distributed under the terms of the Creative Commons Attribution 4.0 International license.

**FIG 3:**
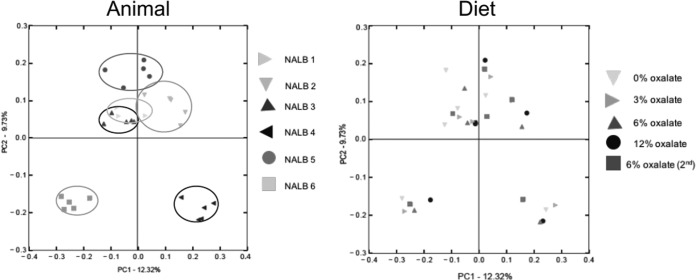
PCoA plots of the β-diversity (unweighted UniFrac) of individuals (left panel [Adonis *P* value of 0.001 and *F* value of 3.402]) and percentage of oxalate in the diet (right panel [Adonis *P* value of 0.632 and *F* value of 0.9526]). Feces were collected 4 to 5 days after the start of each diet treatment for microbial inventories. Different shapes and colors represent different animals or diets. Circles in the left panel show clustering of animals.

**FIG 4:**
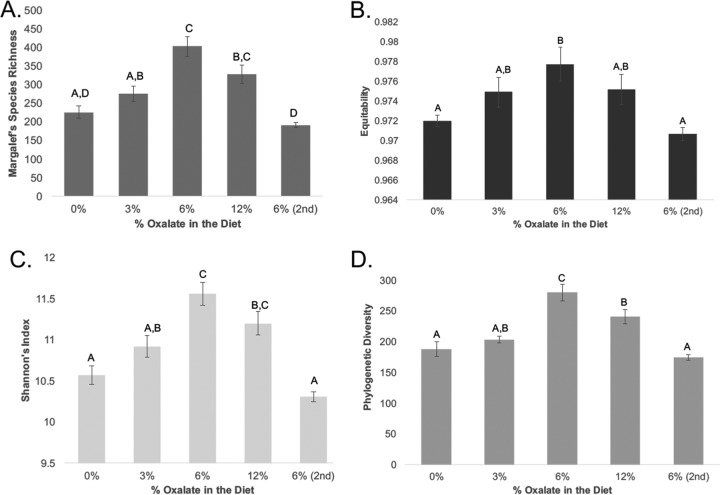
α-Diversity of the gut microbiota in *N. albigula* woodrats fed diets with different concentrations of oxalate. Treatments along the *x* axis are listed chronologically. Different letters indicate significant differences as determined with a Tukey’s *post hoc* analysis. (A) Species richness (repeated-measure ANOVA, df = 5.24, *P* < 0.001); (B) Species evenness (repeated-measure ANOVA, *P* = 0.006); (C) Shannon’s index (repeated-measures ANOVA, *P* < 0.001); (D) phylogenetic diversity (repeated-measures ANOVA, *P* < 0.001).

Community membership and structure were most strongly influenced by interindividual variation (membership, *P* = 0.001, *F* = 3.402; structure, *P* = 0.001, *F* = 5), and not by oxalate treatment (membership, *P* = 0.632, *F* = 0.9526; structure, *P* = 0.059, *F* = 1.43) (Fig. 3). All α-diversity metrics differed significantly across the diet trial, with a peak at the first 6% oxalate period and a trough at the end of the diet trial (Fig. 4). Coinciding with the trend in α-diversity, 8 of the 101 defined families exhibited a significant difference in the number of unique OTUs assigned to them, with a peak at the first 6% oxalate period and trough at the end of the diet trial (Table 2). We conducted paired *t* tests on the relative abundance of OTUs between the end of the first 6% oxalate period and the end of the diet trial to determine which OTUs dropped out of the community after the first 6% oxalate period. Analysis resulted in the identification of over 1,000 OTUs that saw a significant drop in relative abundance. However, significance was lost when correcting for false discoveries, indicative of a stochastic loss of OTUs (data not shown).

**TABLE 2:** Taxonomic families (out of 101) that exhibit significant differences in number of unique OTUs among oxalate diets^a^

Family	No. of OTUs on diet shown:					FDR
	0% oxalate	3% oxalate	6% oxalate	12% oxalate	6% oxalate (2nd)	
*Lactobacillaceae*	19 ± 1.7 A (1.89E−3)	18.8 ± 2.4 C (1.88E−3)	38.3 ± 2.6 B (2.62E−3)	26.5 ± 3.5 A (1.75E−3)	19.2 ± 2.8 A (2.26E−3)	0.001
S24-7	625 ± 155.1 A (1.92E−1)	1,199.5 ± 147.1 BC (4.44E−1)	1,478.2 ± 88.6 C (3.56E−1)	1,402.7 ± 77 C (4.34E−1)	848.2 ± 62.9 AB (3.56E−1)	0.002
*Ruminococcaceae*	510.5 ± 54.1 A (1.09E−1)	450 ± 19.1 A (9.20E−2)	728.7 ± 68.2 B (1.04E−1)	576.8 ± 49.2 AB (1.03E−1)	416.2 ± 30.8 A (1.15E−1)	0.01
*Coriobacteriaceae*	6.2 ± 1 A (3.47E−4)	9.2 ± 1.4 AB (2.82E−4)	14 ± 2.2 B (2.76E−4)	10.7 ± 1.3 AB (2.67E−4)	4.7 ± 1.2 A (1.50E−4)	0.01
*Lachnospiraceae*	382.8 ± 36.6 AB (1.37E−1)	259.8 ± 25.6 BC (5.57E−2)	431 ± 53.3 A (9.35E−2)	299.8 ± 24 ABC (4.74E−2)	234.3 ± 28.8 C (8.08E−2)	0.02
*Streptococcaceae*	7.3 ± 1.5 AB (0.001101295)	10.7 ± 2.2 B (0.000659257)	12.5 ± 1.3 B (0.000714242)	11 ± 0.6 B (0.000589205)	4.8 ± 0.9 A (0.000601328)	0.02
*Rikenellaceae*	68.8 ± 9.3 AB (4.90E−2)	71.3 ± 6.2 AB (3.63E−2)	93 ± 3.7 B (3.85E−2)	90.8 ± 10.5 AB (3.77E−2)	53.7 ± 4.1 A (3.70E−2)	0.03
*Mogibacteriaceae*	6 ± 0.7 A (4.53E−4)	5.7 ± 1.1 A (2.35E−4)	12.8 ± 1.9 B (4.01E−4)	9.2 ± 1.5 AB (2.30E−4)	8.2 ± 1 AB (4.83E−4)	0.03

a Columns reflect the mean number of unique OTUs ± standard error. Significance was calculated with a repeated-measure ANOVA (FDR corrected). Letters reflect statistical grouping as determined by an FDR-corrected, *post hoc* Tukey’s analysis. The relative abundance of each family and time point is listed in parentheses after the unique number of OTUs.

**FIG 5:**
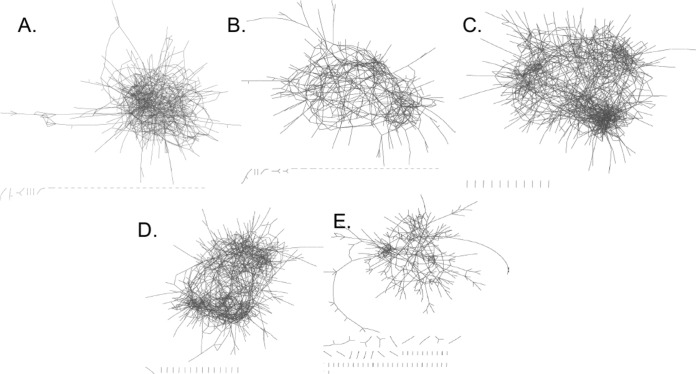
Multilayered cooccurrence network analysis of OTUs that exhibit a significant positive Spearman’s correlation with oxalate consumption. Nodes, which have been minimized to enable clear viewing of the whole network, represent individual OTUs, and edges represent a significant interaction (*P* = 0). Dashes at the bottom of each image reflect isolated interactions between two or a few OTUs. (A) Zero percent oxalate, 1,192 interactions; (B) 3% oxalate, 1,040 interactions; (C) 6% oxalate, 1,682 interactions; (D) 12% oxalate, 1,575 interactions; (E) 6% oxalate (second), 729 interactions.

Repeated-measure Spearman correlations revealed that 1,004 OTUs out of the total 22,784 OTUs present in the six animals analyzed across the study exhibited a significant positive correlation with oxalate consumption after false-discovery rate (FDR) correction, while no OTUs exhibited a significant negative correlation, indicating that equivalent reductions in relative abundance were broadly distributed among the remaining OTUs (see Table S2 in the supplemental material). The SparCC analysis revealed a variable number of positive significant interactions between diet treatments ranging from 729 at the end of the diet trial to 1,682 after the first 6% oxalate period (Fig. 5). Furthermore, there was a significant increase in the relative abundance of the oxalate-degrading genes *oxc* and *frc* (Fig. S4).

10.1128/mSphere.00428-17.6 TABLE S2  Microbial OTUs (out of 22,784) that exhibit a significant positive correlation (repeated-measure Spearman’s correlation) with oxalate intake. Relative abundances were calculated after normalization, and *P* values have been FDR corrected. Download TABLE S2, PDF file, 0.2 MB. Copyright © 2017 Miller et al. 2017 Miller et al. This content is distributed under the terms of the Creative Commons Attribution 4.0 International license.

10.1128/mSphere.00428-17.4 FIG S4  The abundance of the *oxc* (A) and *frc* (B) genes for the fecal microbiota after the 0% oxalate diet compared to after the first 6% oxalate diet. There was a significant increase in abundance for both the *oxc* (*t* = 2.675, df = 5, *P* = 0.047) and *frc* (*t* = 2.879, df = 5, *P* = 0.031) genes. Download FIG S4, PDF file, 0.05 MB. Copyright © 2017 Miller et al. 2017 Miller et al. This content is distributed under the terms of the Creative Commons Attribution 4.0 International license.

### Effect of oxalate on renal histopathology.

**FIG 6:**
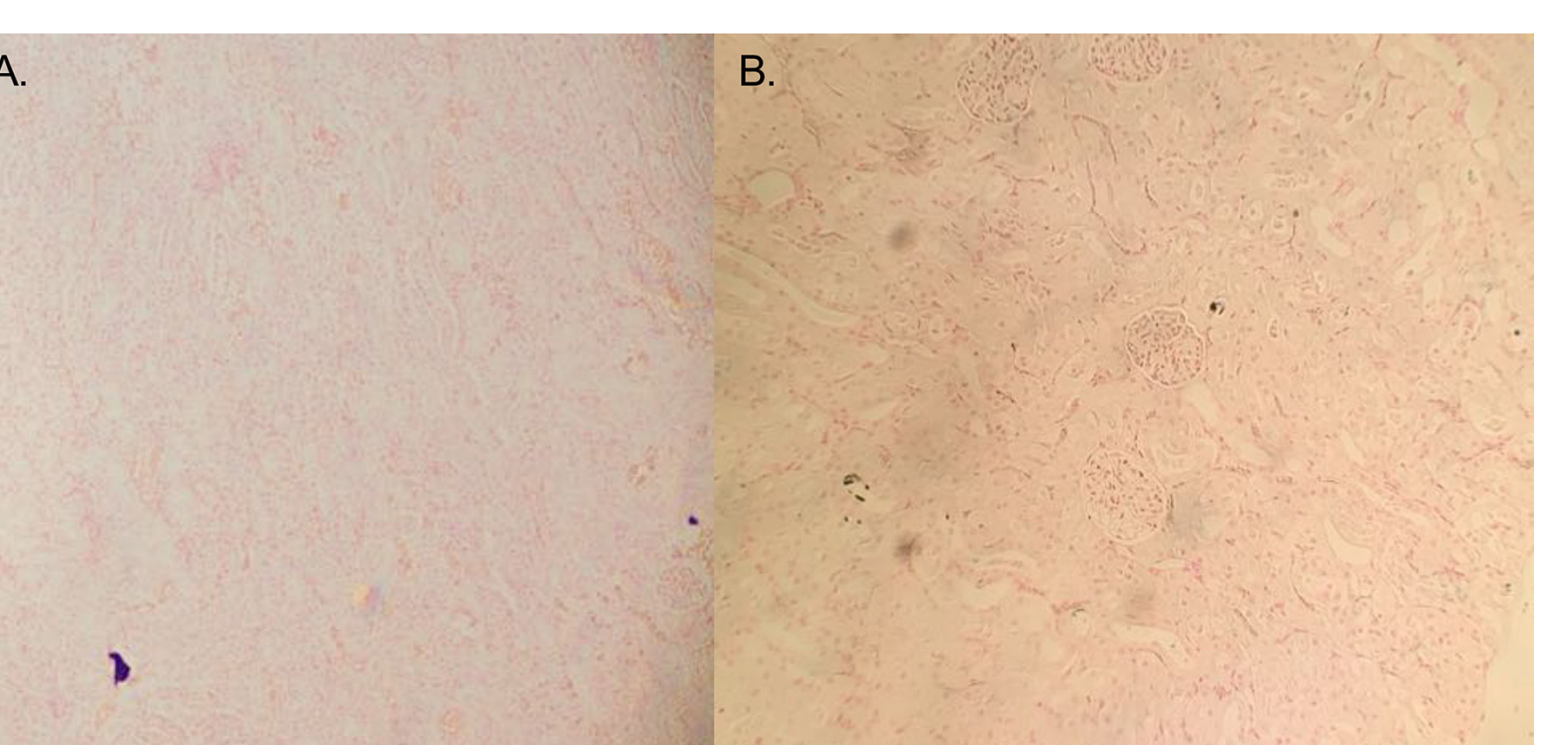
Von Kossa staining of kidney sections from freshly trapped woodrats on a 1.5% oxalate diet (A) and captive animals on a 12% oxalate diet (B). Black areas represent calcium deposits within the tissue.

Renal calcium deposition did not correlate with oxalate consumption in *N. albigula*. There were no significant differences in the surface area of kidney sections normalized to body mass between the wild-diet-fed and high-oxalate-diet-fed animals (Table 3). All kidneys, whether from animals feeding on the wild diet or the high-oxalate diet, exhibited some calcium deposition (Fig. 6). However, there was no significant increase in the percentage of the total kidney surface area covered by calcium deposits for *N. albigula* feeding on a 1.5% oxalate diet versus 6 or 12% oxalate (Table 3).

**TABLE 3:** Surface area of kidney sections normalized to body mass and surface area covered by calcium deposits in *Neotoma albigula* woodrats freshly caught in the wild or given a high-oxalate diet^a^

Metric	Result for woodrat group		*t* score	*P* value
	Wild caught	High-oxalate diet		
Surface area of kidney section (cm^2^/kg body mass)	8.7 ± 1.1	8 ± 0.5	3.955	0.58
% of surface area covered by calcium deposits	0.014 ± 0.007	0.007 ± 0.003	3.722	0.38

a Shown is the surface area of kidney sections normalized to body mass and surface area covered by calcium deposits in *Neotoma albigula* woodrats freshly caught in the wild or given a high-oxalate diet (6 or 12%). Significance was determined by a *t* test (df = 8).

## DISCUSSION

Diet is one of the primary drivers influencing the form and function of the mammalian gut microbiota (25–28). Dietary toxins in particular help to shape and are shaped by the gut microbiota, with considerable implications for the health and physiology of the host (5–8, 19). The results of our study strongly support but do not explicitly confirm the hypothesis that microbial oxalate metabolism in the *N. albigula* gut involves a distinct and cohesive, functionally resilient microbial network with a limited oxalate-buffering capacity. To test the hypothesis, we created artificial conditions for the gut microbiota of *N. albigula* that are well outside what is normally seen in the wild, to specifically stimulate the microbial network that benefits from oxalate exposure and quantify the oxalate-buffering capacity of the gut microbiota as a whole. Prior to the experimental period, animals were under captive conditions, feeding on a very-low-oxalate (0.2%) diet for 7 months. These conditions reduced the gut microbiota α-diversity and helped the microbiota acclimate away from oxalate exposure, thus setting the conditions necessary to quantify the effect of oxalate exposure (7). Following the acclimation period, oxalate, which is one of the primary toxins found in the wild *N. albigula* diet, was reintroduced and brought up to levels 8-fold higher than what is naturally seen (8). Evidence that supports our oxalate metabolic network hypothesis include the following: (i) the microbiota exhibited a two-phase, dynamic change in diversity where oxalate shifted from resource to poison for the gut microbiota, (ii) >1,000 microbial OTUs exhibited a statistically significant positive correlation to oxalate over the course of the diet trial, with the number of interactions among them increasing and decreasing in accordance with oxalate input (Fig. 5), and (iii) despite an overall drop in diversity beginning after the first 6% oxalate period, oxalate metabolism operated at maximum capacity throughout the trial. Each of these points will be discussed in detail below.

Oxalate, from the perspective of the gut microbiota, is both a resource and a poison. On the one hand, oxalate can be used as a carbon and energy source for many OTUs inhabiting the gut, sometimes exclusively, as is the case with *O. formigenes* (8, 14, 29–32). However, oxalate is also known to inhibit the growth of some bacteria that are known oxalate degraders and to induce the differential regulation of over 300 genes (17, 18). In the present study, both aspects of oxalate appear to impact the gut microbiota, inducing a two-phase dynamic shift in the gut microbiota. In the first phase, which lasted up to the first 6% diet, oxalate acted as a resource, enabling the community to support greater diversity (Fig. 4). The increase in diversity may at least partially reflect a return to historical conditions given a reintroduction of a resource with which the gut microbiota had been historically adapted and not just the introduction of a novel resource in general. In previous studies, the gut microbiota has been partially restored in captive animals when their natural diet was reintroduced (7). Similar increases in α-diversity have also occurred with the reintroduction of creosote, a toxin present in the natural diet of other woodrat species, *N. lepida* and *N. bryanti* (33). In contrast, when oxalate or creosote is introduced into the diets of animals without prior experience to the toxins, the gut microbiota exhibits significant decreases in diversity (20, 33). In the second phase of gut microbiota change, which occurs when oxalate reaches 12% of the diet, the level of oxalate exposure exceeds the buffering capacity of the gut microbiota and thus acts as a poison. This level of oxalate consumption is approximately 10× the typical daily consumption for humans (34) (Fig. 2). The effects of oxalate as a poison are reflected in the body mass loss of the *N. albigula* hosts (Fig. 1) and in the α-diversity crash that began at 12% oxalate and persisted to the end of the diet trial (Fig. 4). Additionally, even the *Oxalobacteraceae*, some of which are oxalate-degrading specialists, exhibited a significant decline in relative abundance during the 12% period (see Fig. S2 in the supplemental material). Similar drops in α-diversity have been demonstrated after exposure to antibiotics, which are explicitly used to reduce microbial populations (35, 36).

Over the course of the diet trial, some OTUs exhibited statistically significant changes in relative abundance, while others did not. It is expected that if each animal harbored the exact same microbiota at the start of the trial, then consistent OTU-level shifts in relative abundance would occur and a clear diet effect would be apparent. Indeed, 1,004 out of 22,784 OTUs (3.8%) and 8 out of 101 families (7.9%) exhibited significant positive correlations to oxalate exposure over the course of the experiment (Table 2; and Table S2). However, there were no OTUs that were negatively correlated to oxalate exposure, meaning that the reciprocal declines in relative abundance for other taxa were broadly distributed in a stochastic manner. Our results indicate that the correlation between the relative abundance of OTUs and oxalate consumption followed by cooccurrence analysis is an effective means to identify and track bacteria that respond to oxalate, even with the sample size of six animals. As shown in the principal coordinate analysis (PCoA) plots of β-diversity (Fig. 3), microbial communities clustered significantly by animal, but not by diet. The β-diversity results indicate that the stochastic shifts in OTUs that are dependent on the unique microbiota of each animal at the start of the experiment outweighed the statistically significant OTU responses to oxalate. The taxa that positively responded to oxalate exposure were primarily composed of taxa expected to be a part of an oxalate metabolic network. They included potential oxalate degraders from the *Oxalobacteraceae*, *Lactobacillus*, and *Bifidobacterium*, as well as taxa that harbor a complete oxalate metabolic pathway, such as the S24-7 family (5, 14, 37). In fact, given the number and relative abundance of S24-7 members responding to oxalate, they may be even more important to oxalate degradation than the *Oxalobacteraceae* (Table 2; Table S2). Other taxa that responded to oxalate exposure do not have an obvious association with oxalate metabolism. These bacteria may benefit indirectly from oxalate metabolism, perhaps by taking advantage of the by-products produced, formate and CO_2_. Formate and CO_2_ may be used in downstream pathways such as acetogenesis, methanogenesis, or sulfate reduction (38). Indeed, many of the OTUs with significant correlation to oxalate exposure can potentially engage in these metabolic activities (Table S3). However, it is impossible to determine what functions these bacteria engaged in for the present study. The multilayer network analysis restricted to OTUs that positively correlated to oxalate exposure (Fig. 5) shows a growth of the overall network with increasingly fewer isolated interactions that plateaued between the first 6% and 12% oxalate diets with a collapse by the end of the trial. This is further indication of a cohesive microbial network that specifically responds to oxalate exposure and has a limited buffering capacity. Network analysis was validated by correlating the number of cooccurring pairs at each time point to oxalate degradation. This validation step independently ensures that the endpoint of the network analysis produces biologically meaningful results rather than producing an arbitrary subset of OTUs present in the microbial inventories. As expected, there was a significant correlation with the biggest deviations from the trendline occurring at the two 6% oxalate periods. The physiological relevance of our results was validated through quantitative PCR (qPCR) analysis of the oxalate-degrading genes, *oxc* and *frc*, which increased in relative abundance between the 0% and 6% oxalate periods. For qPCR analysis, primers were designed based on the *oxc* and *frc* genes from the *Oxalobacteraceae*, which exhibited a significant response to oxalate according to microbial inventories generated by 16S rRNA sequencing (Fig. S4).

10.1128/mSphere.00428-17.7 TABLE S3  List of bacteria that exhibit a significant correlation with oxalate consumption in *N. albigula* that may be involved in the secondary metabolism of the by-products of oxalate degradation. Download TABLE S3, PDF file, 0.1 MB. Copyright © 2017 Miller et al. 2017 Miller et al. This content is distributed under the terms of the Creative Commons Attribution 4.0 International license.

Despite the fact that the oxalate-buffering capacity of the microbiota was exceeded, oxalate metabolism continued to operate at maximum capacity during the 12% and the second 6% oxalate periods (Fig. 2). While the proportion of animals that both were removed and exhibited anomalous values for oxalate excretion/degradation was higher than for those animals that did not drop out, the difference was not significant (Table 1). In examining the network analysis, we see also that the number of interactions after the first 6% diet (1,682) is similar to that after the 12% diet (1,575), in contrast to the overall α-diversity crash (Fig. 4). These results are indicative of a functionally resilient microbial network with a high residual capacity for oxalate metabolism.

Over the course of the diet trial, 50% of the animals lost >10% of their body mass (Fig. 1). However, there was no significant change in food intake (Table S1). Furthermore, in contrast to the significant drop in overall α-diversity between the first and second 6% oxalate periods, there were no significant declines in OTU relative abundance, indicative of stochastic population reductions. These results, combined with the persistently high oxalate degradation and microbial network resilience, suggest that the loss of body mass in the animal hosts was not a direct result of oxalate exposure, but rather could result from the stochastic changes in the gut microbiota and their effect on host physiology. However, further examination of the causes behind the mass loss was out of the scope of the experiment, and it is impossible to know for sure given the data.

During the diet trial, oxalate was added to the diet as sodium oxalate, thus in effect creating two stressors to the host and microbiota. While there is considerable evidence for the effect of sodium on host physiology, little is known about its effect on the microbiota (39–41). The animals here exhibited a significant increase in water intake and urine output in accordance with the oxalate diet, indicative of an effect of sodium (Fig. S1). Furthermore, the genus *Allobaculum*, which exhibited a significant correlation to the diet here, also significantly increases in relative abundance with sodium chloride intake (40). However, in mammalian herbivores, sodium is the most limiting mineral for growth and reproduction (42). Sodium is efficiently absorbed, with very little making it to the microbiota of the distal colon (43). Many of the OTUs stimulated by oxalate here have previously been associated with oxalate or negatively associated with kidney stone disease. *Oxalobacter* is an oxalate specialist (5). Bacteria belonging to the taxa *Oxalobacteraceae*, *Bifidobacterium*, *Clostridium*, S24-7, *Lactobacillus*, and *Clostridiales* are known to harbor oxalate-degrading genes (8, 37). Additionally, bacteria from the *Oxalobacteraceae*, *Parabacteroides*, *Desulfovibrio*, and *Bacteroides* taxa have been negatively associated with kidney stone disease (12, 44, 45). Finally, the taxa stimulated by oxalate here exhibited an 82% overlap with taxa that cooccur with *Oxalobacter* at the family level in woodrats and Sprague-Dawley rats, as determined in another study (46). Therefore, while some of the changes to bacterial taxa may result from exposure to sodium, our results suggest that the changes to the microbiota in this study were primarily driven by oxalate.

Our results indicate that the physiology of the host-microbe holobiont of *N. albigula* is sufficient to inhibit the negative effects of oxalate consumption, even at extreme levels. A diet of 12% oxalate is lethal for many mammals, including cattle and sheep (47). However, even on a 6% or 12% oxalate diet, there was no significant accumulation of renal calcium deposition in *N. albigula*. This is in contrast to Sprague-Dawley rats fed a high-glyoxalate diet, which produces excess endogenous oxalate, which exhibited significant increases in renal calcium deposition and the development of renal calculi (48).

### Conclusions.

The microbiota of *N. albigula* is unique in its capacity for oxalate metabolism, which can be exploited for the development of personalized bacteriotherapies. The results of the present study have broad implications for the development of bacteriotherapies that target oxalate for elimination. Here we have shown that (i) cohesive microbial networks respond to a specific oxalate challenge, (ii) stochastic effects on the microbial community unique to each individual’s microbiota can mask the effect of oxalate on the microbial networks, (iii) the buffering capacity of the *N. albigula* microbiota can be exceeded given a high enough level of oxalate exposure, and (iv) the *N. albigula* oxalate metabolic network is resilient even despite an overall community-level crash. This work can inform strategies for the development of personalized bacteriotherapies for patients suffering from recurrent episodes of calcium oxalate stone formation. Furthermore, the methods employed here can be used to identify microbial networks responsive to other toxins, along with their limits. Thus, this work adds to the toolbox for the development of targeted bacteriotherapies.

## MATERIALS AND METHODS

### Oxalate diet trials.

Fourteen adult *N. albigula* woodrats were collected from Castle Valley, UT (38.63′N, 109.41′W) in September 2014, using Sherman live traps. Animals were transported to the animal facility at University of Utah, where they were housed in individual cages (48 by 27 by 20 cm) with a 12-h/12-h light/dark cycle, at 28°C and 20% humidity. Animals were maintained on a 0.2% oxalate, high-fiber rabbit chow (Harlan Teklad formula 2031; Envigo, Denver, CO) for 7 months prior to experimentation, which reduces the overall diversity of the microbiota of these animals but maintains their native microbiota (49). Thus, captive maintenance on a low-oxalate diet provides a baseline microbiota to which the addition of oxalate can be compared. All methods were approved by the IACUC under protocol no. 12-12010.

Woodrats were placed in a diet trial in which the oxalate concentration of the food was gradually increased over time along a gradient from 0.2% (for days 1 to 5; herein referred to as 0%), 3% (days 6 to 10), 6% (days 11 to 15), 12% (days 16 to 30 days), and then decreased to 6% (days 31 to 36) (Table 4). The oxalate concentration of the diet was adjusted by adding the appropriate amount of sodium oxalate (Fisher Scientific, Pittsburgh, PA) into the powdered rabbit chow on a dry weight basis. Animals were removed from the diet trial if they lost >10% of their starting body mass. The maximum tolerable dose of oxalate was defined by the point at which 50% of the animals were removed from the diet trial due to body mass loss. The multiday schedule for each concentration was chosen to ensure that the gut microbiota had time to respond to the specific diet (19, 50). The 14-day period for the 12% oxalate diet was employed to evaluate the potential for long-term persistence at a high concentration of oxalate. We did not go above 12%, as this concentration is known to be lethal to other mammalian herbivores and is found in some plants native to the same habitat as *N. albigula* (47). During the trial, we measured body mass and food intake on a daily basis, and from these data we estimated DMD and oxalate consumption.

**TABLE 4:** Timeline for the diet trial to quantify the maximum tolerable dose of oxalate and its effect on the gut microbiota

Diet	No. of days on diet	Day of fecal collection^a^
0% oxalate	5	5
3% oxalate	4	4
6% oxalate	5	5
12% oxalate	14	4
6% oxalate (2nd)	5	5

a Feces were collected for microbial inventories at the specified time points.

For the duration of the diet trial, animals were placed in metabolic cages to separate urine and feces into sterile 50-ml conical tubes to allow for quantification along with food and water intake (given *ad libitum*). Acidified 24-h urine collections were frozen (−20°C) and feces dried overnight (45°C) prior to oxalate assays. Prior to drying, a subsample of feces was collected from each animal every 4 to 5 days on each diet treatment. Samples were frozen at −80°C for microbial analyses. Oxalate excretion was also quantified from urine and feces for the same time periods (Table 4).

Oxalate extractions and assays were performed previously described (19, 20). Briefly, acidified urine was thawed and centrifuged to remove precipitates. The pH of the urine was brought up to 7 with NaOH, and 0.1 g of CaCl_2_ was added to precipitate calcium oxalate. Samples were centrifuged and decanted. A volume of deionized water equal to the starting volume of urine was added to calcium oxalate precipitate, and the solution was titrated as described below. Dried fecal samples were ground to a powder and acidified with H_2_SO_4_ for 15 min to extract oxalate. Precipitates were filtered out with a grade 4 Whatman filter, and the pH was raised to 7 with NaOH. Calcium oxalate was precipitated with the addition of CaCl_2_, and a volume of deionized water equal to the volume of the filtrate was added prior to titration. Calcium oxalate solutions were acidified and heated to 80°C prior to titration with 0.01 M KMnO_4_. Titration volumes were then compared to standards that have undergone the same procedures with known amounts of oxalate added. These methods allow for the recovery of 90 to 110% of urinary and fecal oxalate (19, 20). Oxalate degradation was defined as oxalate consumed minus oxalate excreted, which is a conservative estimate (19, 20). Data were evaluated with repeated-measure analysis of variance (ANOVA) with a *post hoc* Tukey’s analysis when applicable of animals that made it to the end of the trial. To determine if animals that were removed from the trial for excessive loss of mass exhibited a difference in oxalate degradation or urinary oxalate excretion from those that persisted, box and whisker plots were generated from all data for each time point and anomalous values were defined as being outside 1.5× the interquartile range above the upper quartile or below the lower quartile. The proportions of anomalous values in each group were compared with a Fisher’s exact test.

### Microbial inventories.

For microbial inventories, DNA was extracted from 180 to 220 mg of fecal samples with the QIAamp DNA stool minikit (Qiagen, Germantown, MD). Microbial inventories were generated from feces of the seven woodrats that persisted through the entire trial. Extracted DNA was sent to Argonne National Laboratory (Chicago, IL) for sequencing of the V4 region of the 16S rRNA gene with primers 515F and 806R (51). The DNA was barcoded with 12-bp sequences, and samples were multiplexed for a single-lane run on an Illumina MiSeq with paired-end sequencing of 150 bp each, as described previously (52).

The resulting data were demultiplexed and quality controlled with default parameters in QIIME (52). Microbial OTUs were assigned *de novo* with UCLUST at a sequence identity cutoff of 97%. Chloroplast and mitochondrion sequences, as well as sequences with fewer than 10 representations across the whole data set, were removed. Following processing, the data set was normalized with the DESEQ2 algorithm, which executes a negative binomial Wald test and maintains rare taxa (53, 54).

From the normalized OTU table, we calculated the following α-diversity metrics: species richness (Margalef’s), evenness (equitability), Shannon index, and phylogenetic diversity. The α-diversity metrics were analyzed with a repeated-measure ANOVA and *post hoc* Tukey’s analysis or a paired *t* test with false-discovery rate (FDR) correction where applicable. Furthermore, unweighted and weighted UniFrac analyses were performed to compare community membership and structure, respectively (55). Comparisons between animals and diet were made with Adonis after 999 permutations. The total number of unique OTUs in each bacterial family for each diet was quantified and analyzed with a repeated-measure ANOVA across time points (FDR corrected) followed by a *post hoc* Tukey’s analysis (R statistical package). All analyses were performed in QIIME unless otherwise noted.

Those OTUs exhibiting a significant increase in relative abundance we defined as an oxalate metabolic network, which was specifically tracked across the diet trial. To quantify the network, we performed a repeated-measure Spearman correlation analysis (FDR corrected) for OTU relative abundance and oxalate consumption using R statistical software. Spearman correlations were chosen because they are more appropriate for nonlinear correlations, and we expected a plateauing or decline of microbial network bacteria at the 12% oxalate level. Significant, positive Spearman correlations were used to produce a restricted list of OTUs. From this list, the SparCC algorithm was used for cooccurrence analyses of the OTUs among animals and time points. This algorithm minimizes spurious associations and only strong correlations (*P* = 0) were used in downstream analyses (56). Cooccurrence networks were visualized in Cytoscape (57).

### Validation of network analysis.

Network analysis results were validated in two ways. First, the number of cooccurring pairs within the network was quantified for presence/absence in the microbiota of each animal and time point. This was then correlated to oxalate degradation at the same time point. This ensures that the biological significance inferred from network analysis correlated to the expected biological outcomes and that the analytical methods to identify OTUs responding to oxalate were valid. Second, we conducted qPCR analysis of the oxalate-degrading genes *oxc* and *frc* for the microbiota after the 0.2% oxalate diet and after the first 6% oxalate diet, where the biggest difference in microbial diversity was quantified. This analysis ensures that the microbial function expected to increase the most between these time points is genetically validated. For qPCR analysis, primers were designed using genomes from the *Oxalobacteraceae* family. Specifically, oxalate-degrading genes *oxc* (forward primer 5′ CACGTCCACATTGTAAAGCA 3′ and reverse primer 5′ ACCTGGACGACCTGAAACTG 3′) and *frc* (forward primer 5′ TTCAGGTGCAGCATTAGGTG 3′ and reverse primer 5′ ACCACGACCAGTTTTATGACG 3′) were quantified. Extracted DNA was normalized to the same concentration, and qPCR was performed on an Applied Biosystems StepOne Plus using the SYBR green qPCR master mix (Roche). The thermal cycling conditions were as follows: 95°C for 10 min, followed by 40 amplification cycles (95°C for 15 s and 52°C for 60 s) and a slow ramp up to 95°C for dissociation. A standard curve was made for the *oxc* and *frc* genes using purified PCR product and ranged from 10^1^ to 10^5^ gene copies. All reactions were performed in triplicate on the same 96-well plate for each gene. Gene copy numbers in the samples were automatically determined by comparison of the threshold cycle (*C*_*T*_) values to the standard curve (*R*^2^ > 0.97) in the OneStep software.

### Histopathology.

A total of eight *N. albigula* woodrats were euthanized under isoflurane for the histopathological analysis of kidneys. As a control, four animals in captivity for one night after trapping while feeding on their natural diet of *Opunita* cactus (1.5% oxalate) were sacrificed to quantify normal renal calcium deposition for *N. albigula*. To maximize the probability of detecting renal calcium oxalate deposition for animals in the present study, two animals that dropped out while on 6% oxalate and two animals that dropped out on 12% oxalate were chosen for renal histopathology. Kidneys were fixed in formalin, which was replaced with 70% ethanol after 24 h and kept at 4°C until they were processed for histology. Prior to embedding in paraffin, kidneys were longitudinally bisected along the center line of the kidney. Longitudinal sections of the center of the kidney were stained with the Von Kossa stain, in which silver nitrate is used to replace calcium and becomes opaque. Kidney sections were visualized on an Olympus BX41 microscope at ×100 magnification. Images were taken with a Diagnostic Instruments color mosaic camera (18.2) and Spot version 4.6 software. Total kidney surface area and the surface area of calcium deposits were quantified with ImageJ software.

### **Accession number**(s)**.**

Sequence reads are available at the Sequence Read Archive under accession no. SRR5261472.

## References

[1] Qin J, Li R, Raes J, Arumugam M, Burgdorf KS, Manichanh C, Nielsen T, Pons N, Levenez F, Yamada T, Mende DR, Li J, Xu J, Li S, Li D, Cao J, Wang B, Liang H, Zheng H, Xie Y, Tap J, Lepage P, Bertalan M, Batto JM, Hansen T, Le Paslier D, Linneberg A, Nielsen HB, Pelletier E, Renault P, Sicheritz-Ponten T, Turner K, Zhu H, Yu C, Li S, Jian M, Zhou Y, Li Y, Zhang X, Li S, Qin N, Yang H, Wang J, Brunak S, Dore J, Guarner F, Kristiansen K, Pedersen O, Parkhill J, Weissenbach J, Bork P, Enrlich SD, Wang J 2010 A human gut microbial gene catalogue established by metagenomic sequencing. Nature 464:59–65. doi:10.1038/nature08821. 20203603 PMC3779803

[2] Hooper LV, Littman DR, Macpherson AJ 2012 Interactions between the microbiota and the immune system. Science 336:1268–1273. doi:10.1126/science.1223490. 22674334 PMC4420145

[3] Nicholson JK, Holmes E, Kinross J, Burcelin R, Gibson G, Jia W, Pettersson S 2012 Host-gut microbiota metabolic interactions. Science 336:1262–1267. doi:10.1126/science.1223813. 22674330

[4] Sommer F, Bäckhed F 2013 The gut microbiota—masters of host development and physiology. Nat Rev Microbiol 11:227–238. doi:10.1038/nrmicro2974. 23435359

[5] Allison MJ, Dawson KA, Mayberry WR, Foss JG 1985 *Oxalobacter formigenes* gen. nov., sp. nov.: oxalate-degrading anaerobes that inhabit the gastrointestinal tract. Arch Microbiol 141:1–7. doi:10.1007/BF00446731. 3994481

[6] Jones RJ, Megarrity RG 1986 Successful transfer of DHP-degrading bacteria from Hawaiian goats to Australian ruminants to overcome the toxicity of Leucaena. Aust Vet J 63:259–262. doi:10.1111/j.1751-0813.1986.tb02990.x. 3790013

[7] Kohl KD, Weiss RB, Cox J, Dale C, Dearing MD 2014 Gut microbes of mammalian herbivores facilitate intake of plant toxins. Ecol Lett 17:1238–1246. doi:10.1111/ele.12329. 25040855

[8] Miller AW, Oakeson KF, Dale C, Dearing MD 2016 Microbial community transplant results in increased and long-term oxalate degradation. Microb Ecol 72:470–478. doi:10.1007/s00248-016-0800-2. 27312892 PMC5155304

[9] Noonan SC, Savage GP 1999 Oxalate content of foods and its effect on humans. Asia Pac J Clin Nutr 8:64–74. doi:10.1046/j.1440-6047.1999.00038.x. 24393738

[10] Franceschi VR, Nakata PA 2005 Calcium oxalate in plants: formation and function. Annu Rev Plant Biol 56:41–71. doi:10.1146/annurev.arplant.56.032604.144106. 15862089

[11] Moe OW 2006 Kidney stones: pathophysiology and medical management. Lancet 367:333–344. doi:10.1016/S0140-6736(06)68071-9. 16443041

[12] Kaufman DW, Kelly JP, Curhan GC, Anderson TE, Dretler SP, Preminger GM, Cave DR 2008 *Oxalobacter formigenes* may reduce the risk of calcium oxalate kidney stones. J Am Soc Nephrol 19:1197–1203. doi:10.1681/ASN.2007101058. 18322162 PMC2396938

[13] Hodgkinson A 1977 Oxalic acid in biology and medicine. Academic Press, London, United Kingdom.

[14] Turroni S, Vitali B, Bendazzoli C, Candela M, Gotti R, Federici F, Pirovano F, Brigidi P 2007 Oxalate consumption by lactobacilli: evaluation of oxalyl-CoA decarboxylase and formyl-CoA transferase activity in *Lactobacillus acidophilus*. J Appl Microbiol 103:1600–1609. doi:10.1111/j.1365-2672.2007.03388.x. 17953571

[15] Baetz AL, Allison MJ 1989 Purification and characterization of oxalyl-coenzyme A decarboxylase from *Oxalobacter formigenes*. J Bacteriol 171:2605–2608. doi:10.1128/jb.171.5.2605-2608.1989. 2708315 PMC209940

[16] Baetz AL, Allison MJ 1990 Purification and characterization of formyl-coenzyme A transferase from *Oxalobacter formigenes*. J Bacteriol 172:3537–3540. doi:10.1128/jb.172.7.3537-3540.1990. 2361939 PMC213325

[17] Campieri C, Campieri M, Bertuzzi V, Swennen E, Matteuzzi D, Stefoni S, Pirovano F, Centi C, Ulisse S, Famularo G, De Simone C 2001 Reduction of oxaluria after an oral course of lactic acid bacteria at high concentration. Kidney Int 60:1097–1105. doi:10.1046/j.1523-1755.2001.0600031097.x. 11532105

[18] Azcarate-Peril MA, Bruno-Bárcena JM, Hassan HM, Klaenhammer TR 2006 Transcriptional and functional analysis of oxalyl-coenzyme A (CoA) decarboxylase and formyl-CoA transferase genes from *Lactobacillus acidophilus*. Appl Environ Microbiol 72:1891–1899. doi:10.1128/AEM.72.3.1891-1899.2006. 16517636 PMC1393175

[19] Miller AW, Kohl KD, Dearing MD 2014 The gastrointestinal tract of the white-throated woodrat (*Neotoma albigula*) harbors distinct consortia of oxalate-degrading bacteria. Appl Environ Microbiol 80:1595–1601. doi:10.1128/AEM.03742-13. 24362432 PMC3957601

[20] Miller AW, Oakeson KF, Dale C, Dearing MD 2016 Effect of dietary oxalate on the gut microbiota of the mammalian herbivore *Neotoma albigula*. Appl Environ Microbiol 82:2669–2675. doi:10.1128/AEM.00216-16. 26896138 PMC4836426

[21] Conyers RA, Bais R, Rofe AM 1990 The relation of clinical catastrophes, endogenous oxalate production, and urolithiasis. Clin Chem 36:1717–1730. 2208646

[22] Hatch M, Cornelius J, Allison M, Sidhu H, Peck A, Freel RW 2006 *Oxalobacter* sp. reduces urinary oxalate excretion by promoting enteric oxalate secretion. Kidney Int 69:691–698. doi:10.1038/sj.ki.5000162. 16518326

[23] Shirley EK, Schmidt-Nielsen K 1967 Oxalate metabolism in the pack rat, sand rat, hamster, and white rat. J Nutr 91:496–502. 6043651 10.1093/jn/91.4.496

[24] Justice KE 1985 Oxalate digestibility in *Neotoma albigula* and *Neotoma mexicana*. Oecologia 67:231–234. doi:10.1007/BF00384290. 28311315

[25] Ley RE, Hamady M, Lozupone C, Turnbaugh PJ, Ramey RR, Bircher JS, Schlegel ML, Tucker TA, Schrenzel MD, Knight R, Gordon JI 2008 Evolution of mammals and their gut microbes. Science 320:1647–1651. doi:10.1126/science.1155725. 18497261 PMC2649005

[26] Turnbaugh PJ, Ridaura VK, Faith JJ, Rey FE, Knight R, Gordon JI 2009 The effect of diet on the human gut microbiome: a metagenomic analysis in humanized gnotobiotic mice. Sci Transl Med 1:6ra14. doi:10.1126/scitranslmed.3000322. PMC2894525 20368178

[27] De Filippo C, Cavalieri D, Di Paola M, Ramazzotti M, Poullet JB, Massart S, Collini S, Pieraccini G, Lionetti P 2010 Impact of diet in shaping gut microbiota revealed by a comparative study in children from Europe and rural Africa. Proc Natl Acad Sci U S A 107:14691–14696. doi:10.1073/pnas.1005963107. 20679230 PMC2930426

[28] Muegge BD, Kuczynski J, Knights D, Clemente JC, González A, Fontana L, Henrissat B, Knight R, Gordon JI 2011 Diet drives convergence in gut microbiome functions across mammalian phylogeny and within humans. Science 332:970–974. doi:10.1126/science.1198719. 21596990 PMC3303602

[29] Hokama S, Honma Y, Toma C, Ogawa Y 2000 Oxalate-degrading *Enterococcus faecalis*. Microbiol Immunol 44:235–240. doi:10.1111/j.1348-0421.2000.tb02489.x. 10832966

[30] Holmes RP, Goodman HO, Assimos DG 2001 Contribution of dietary oxalate to urinary oxalate excretion. Kidney Int 59:270–276. doi:10.1046/j.1523-1755.2001.00488.x. 11135080

[31] Sahin N, Gokler I, Tamer AU 2002 Isolation, characterization and numerical taxonomy of novel oxalate-oxidizing bacteria. J Microbiol 40:109–118.

[32] Weese JS, Weese HE, Rousseau J 2009 Identification of *Oxalobacter formigenes* in the faeces of healthy cats. Lett Appl Microbiol 49:800–802. doi:10.1111/j.1472-765X.2009.02722.x. 19780961

[33] Kohl KD, Dearing MD 2012 Experience matters: prior exposure to plant toxins enhances diversity of gut microbes in herbivores. Ecol Lett 15:1008–1015. doi:10.1111/j.1461-0248.2012.01822.x. 22715970

[34] Jaeger P, Robertson WG 2004 Role of dietary intake and intestinal absorption of oxalate in calcium stone formation. Nephron Physiol 98:p64–p71. doi:10.1159/000080266. 15499217

[35] Dethlefsen L, Huse S, Sogin ML, Relman DA 2008 The pervasive effects of an antibiotic on the human gut microbiota, as revealed by deep 16S rRNA sequencing. PLoS Biol 6:e280. doi:10.1371/journal.pbio.0060280. 19018661 PMC2586385

[36] Dethlefsen L, Relman DA 2011 Incomplete recovery and individualized responses of the human distal gut microbiota to repeated antibiotic perturbation. Proc Natl Acad Sci U S A 108:4554–4561. doi:10.1073/pnas.1000087107. 20847294 PMC3063582

[37] Ormerod KL, Wood DLA, Lachner N, Gellatly SL, Daly JN, Parsons JD, Dal’Molin CGO, Palfreyman RW, Nielsen LK, Cooper MA, Morrison M, Hansbro PM, Hugenholtz P 2016 Genomic characterization of the uncultured Bacteroidales family S24-7 inhabiting the guts of homeothermic animals. Microbiome 4:36. doi:10.1186/s40168-016-0181-2. 27388460 PMC4936053

[38] Drake HL 2012, Acetogenesis. Springer Science and Business Media, Berlin, Germany.

[39] Baudrand R, Campino C, Carvajal CA, Olivieri O, Guidi G, Faccini G, Vöhringer PA, Cerda J, Owen G, Kalergis AM, Fardella CE 2014 High sodium intake is associated with increased glucocorticoid production, insulin resistance and metabolic syndrome. Clin Endocrinol 80:677–684. doi:10.1111/cen.12225. 23594269

[40] Wilck N, Olesen S, Matus M, Balogh A, Dechend R, Alm E, Muller DN 2014 A high-salt diet alters the composition of intestinal microbiota in mice. Hypertension 64(Suppl 1):A321.

[41] Leonberg-Yoo AK, Sarnak MJ 2017 Don’t pass the salt: evidence to support avoidance of high salt intake in CKD. Am J Kidney Dis 69:175–178. doi:10.1053/j.ajkd.2016.09.008. 27789126 PMC6034177

[42] Hellgren EC, Pitts WJ 1997 Sodium economy in white-tailed deer ( Odocoileus virginianus ). Physiol Zool 70:547–555. doi:10.1086/515861. 9279921

[43] Michell A 2014 The clinical biology of sodium: the physiology and pathophysiology of sodium in mammals. Elsevier, Philadelphia, PA.

[44] Gnanandarajah JS, Johnson TJ, Kim HB, Abrahante JE, Lulich JP, Murtaugh MP 2012 Comparative faecal microbiota of dogs with and without calcium oxalate stones. J Appl Microbiol 113:745–756. doi:10.1111/j.1365-2672.2012.05390.x. 22788835

[45] Stern JM, Moazami S, Qiu Y, Kurland I, Chen Z, Agalliu I, Burk R, Davies KP 2016 Evidence for a distinct gut microbiome in kidney stone formers compared to non-stone formers. Urolithiasis 44:399–407. doi:10.1007/s00240-016-0882-9. 27115405 PMC8887828

[46] Miller AW, Dale C, Dearing MD 2017 The induction of oxalate metabolism *in vivo* is more effective with functional microbial communities than with functional microbial species. mSystems 2:e00088-17. doi:10.1128/mSystems.00088-17. 28951890 PMC5613171

[47] James LF, Butcher JE 1972 Halogeton poisoning of sheep: effect of high level oxalate intake. J Anim Sci 35:1233–1238. doi:10.2527/jas1972.3561233x. 4647453

[48] Yamaguchi S, Wiessner JH, Hasegawa AT, Hung LY, Mandel GS, Mandel NS 2005 Study of a rat model for calcium oxalate crystal formation without severe renal damage in selected conditions. Int J Urol 12:290–298. doi:10.1111/j.1442-2042.2005.01038.x. 15828958

[49] Kohl KD, Dearing MD 2014 Wild-caught rodents retain a majority of their natural gut microbiota upon entrance into captivity. Environ Microbiol Rep 6:191–195. doi:10.1111/1758-2229.12118. 24596293

[50] Belenguer A, Ben Bati MB, Hervás G, Toral PG, Yáñez-Ruiz DR, Frutos P 2013 Impact of oxalic acid on rumen function and bacterial community in sheep. Animal 7:940–947. doi:10.1017/S1751731112002455. 23298534

[51] Caporaso JG, Lauber CL, Walters WA, Berg-Lyons D, Huntley J, Fierer N, Owens SM, Betley J, Fraser L, Bauer M, Gormley N, Gilbert JA, Smith G, Knight R 2012 Ultra-high-throughput microbial community analysis on the Illumina HiSeq and MiSeq platforms. ISME J 6:1621–1624. doi:10.1038/ismej.2012.8. 22402401 PMC3400413

[52] Caporaso JG, Kuczynski J, Stombaugh J, Bittinger K, Bushman FD, Costello EK, Fierer N, Peña AG, Goodrich JK, Gordon JI, Huttley GA, Kelley ST, Knights D, Koenig JE, Ley RE, Lozupone CA, McDonald D, Muegge BD, Pirrung M, Reeder J, Sevinsky JR, Turnbaugh PJ, Walters WA, Widmann J, Yatsunenko T, Zaneveld J, Knight R 2010 QIIME allows analysis of high-throughput community sequencing data. Nat Methods 7:335–336. doi:10.1038/nmeth.f.303. 20383131 PMC3156573

[53] Love MI, Huber W, Anders S 2014 Moderated estimation of fold change and dispersion for RNA-seq data with DESeq2. Genome Biol 15:550. doi:10.1186/s13059-014-0550-8. 25516281 PMC4302049

[54] McMurdie PJ, Holmes S 2014 Waste not, want not: why rarefying microbiome data is inadmissible. PLoS Comput Biol 10:e1003531. doi:10.1371/journal.pcbi.1003531. 24699258 PMC3974642

[55] Lozupone C, Hamady M, Knight R 2006 UniFrac—an online tool for comparing microbial community diversity in a phylogenetic context. BMC Bioinformatics 7:731. doi:10.1186/1471-2105-7-371. PMC1564154 16893466

[56] Friedman J, Alm EJ 2012 Inferring correlation networks from genomic survey data. PLoS Comput Biol 8:e1002687. doi:10.1371/journal.pcbi.1002687. 23028285 PMC3447976

[57] Shannon P, Markiel A, Ozier O, Baliga NS, Wang JT, Ramage D, Amin N, Schwikowski B, Ideker T 2003 Cytoscape: a software environment for integrated models of biomolecular interaction networks. Genome Res 13:2498–2504. doi:10.1101/gr.1239303. 14597658 PMC403769

