# Supplementary material for: Microbiota Diversification and Crash Induced by Dietary Oxalate in the Mammalian Herbivore Neotoma albigula
Source: mSphere. 2017 Oct 18;2(5):e00428-17. doi: 10.1128/mSphere.00428-17 (PMC5646245; doi:10.1128/mSphere.00428-17)
Supplement: TABLE S1 [file sph005172383st5.pdf]

Metric	Mean	df	F	P
Body mass (g)	168.41 +/- 2.97	4,10	0.69	0.56
Food intake (g)/kg body mass	89.79 +/- 2.36	4,10	1.25	0.31
Fecal output (g)/kg body mass	9.23 +/- 0.53	4,10	1.84	0.15
Fecal oxalate (umols/day)	790.04 +/- 54.19	4,10	1.84	0.15
Urine oxalate (umols/day)	17.88 +/- 2.09	4,10	0.84	0.53
DMD	89.08 +/- 0.56%	4,10	2.62	0.08
