# Supplementary material for: Microbiota Diversification and Crash Induced by Dietary Oxalate in the Mammalian Herbivore Neotoma albigula
Source: mSphere. 2017 Oct 18;2(5):e00428-17. doi: 10.1128/mSphere.00428-17 (PMC5646245; doi:10.1128/mSphere.00428-17)
Supplement: FIG S1 [file sph005172383sf1.pdf]

Figure S1.

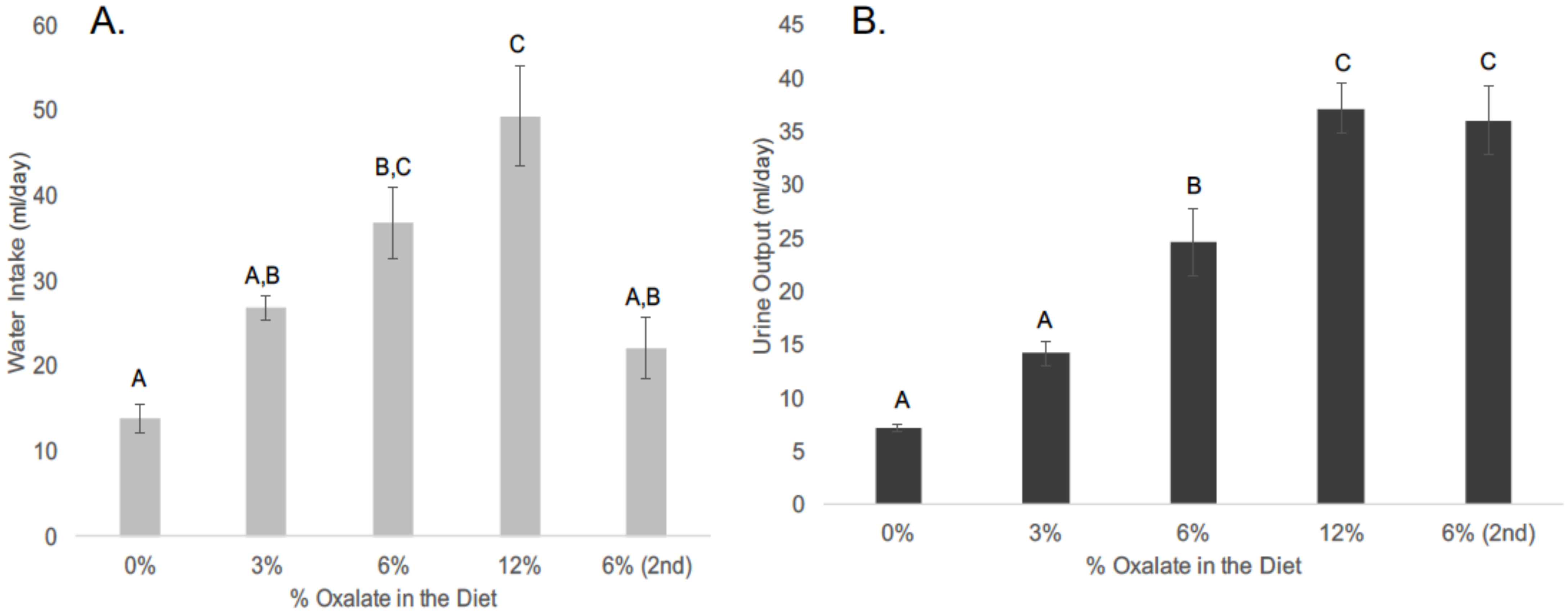

Figure S1. Water intake and urine output for each dietary oxalate treatment. Data for whole experiment was analyzed with a repeated measures ANOVA. Letters indicate statistical groupings determined by a post-hoc Tukey's analysis. A) Water intake (df=6,28, p-value <0.001); B) Urine output (df=6,28, p-value <0.001).
