# Supplementary material for: Microbiota Diversification and Crash Induced by Dietary Oxalate in the Mammalian Herbivore Neotoma albigula
Source: mSphere. 2017 Oct 18;2(5):e00428-17. doi: 10.1128/mSphere.00428-17 (PMC5646245; doi:10.1128/mSphere.00428-17)
Supplement: FIG S2 [file sph005172383sf2.pdf]

Figure S2.

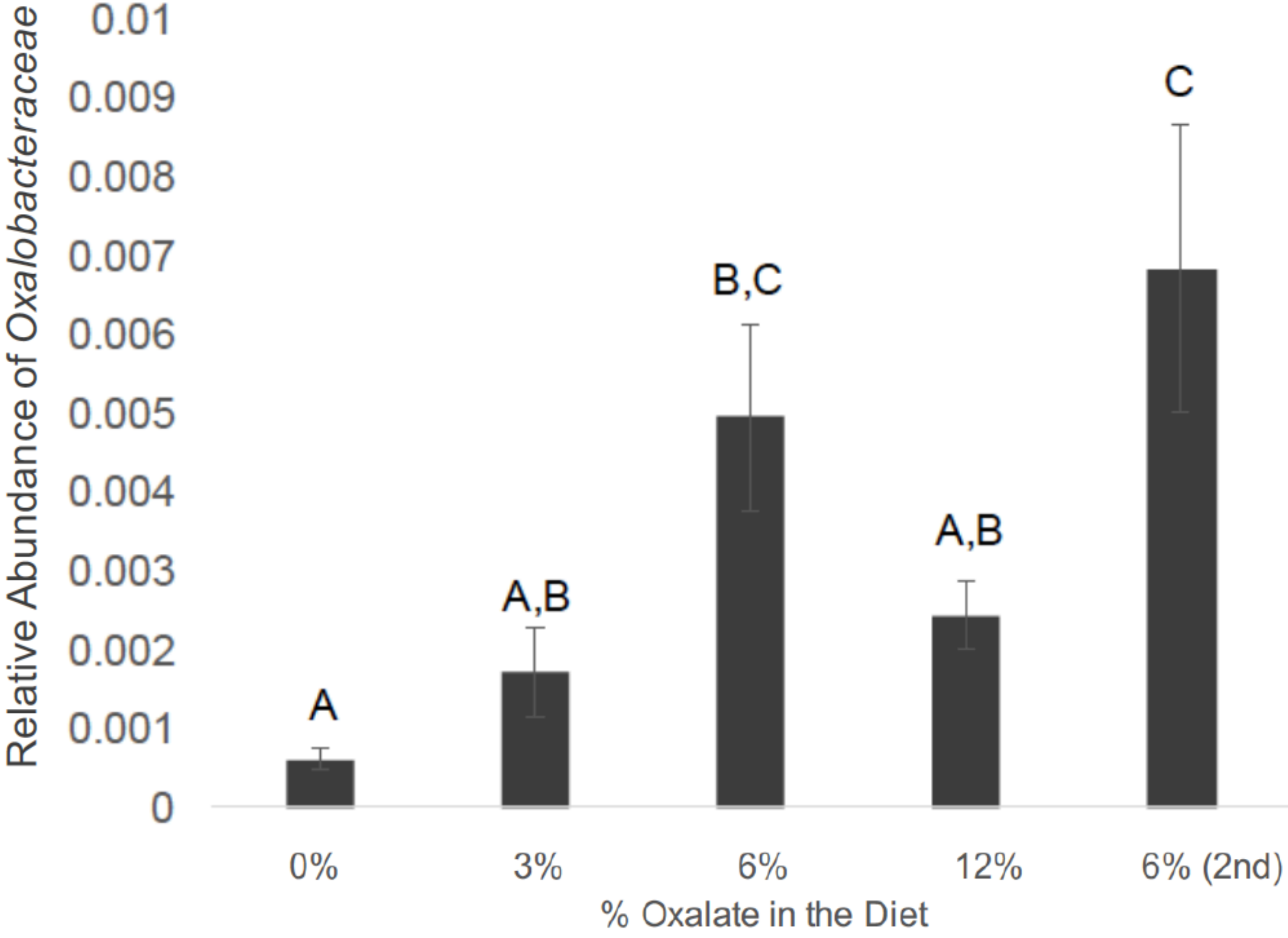

Figure S2. The relative abundance of *Oxalobacteraceae.* Relative abundance was evaluated with a repeated measures ANOVA (5,24) F-value = 4.9198, p-value = 0.006).
