# Supplementary material for: Microbiota Diversification and Crash Induced by Dietary Oxalate in the Mammalian Herbivore Neotoma albigula
Source: mSphere. 2017 Oct 18;2(5):e00428-17. doi: 10.1128/mSphere.00428-17 (PMC5646245; doi:10.1128/mSphere.00428-17)
Supplement: FIG S3 [file sph005172383sf3.pdf]

Figure S3.

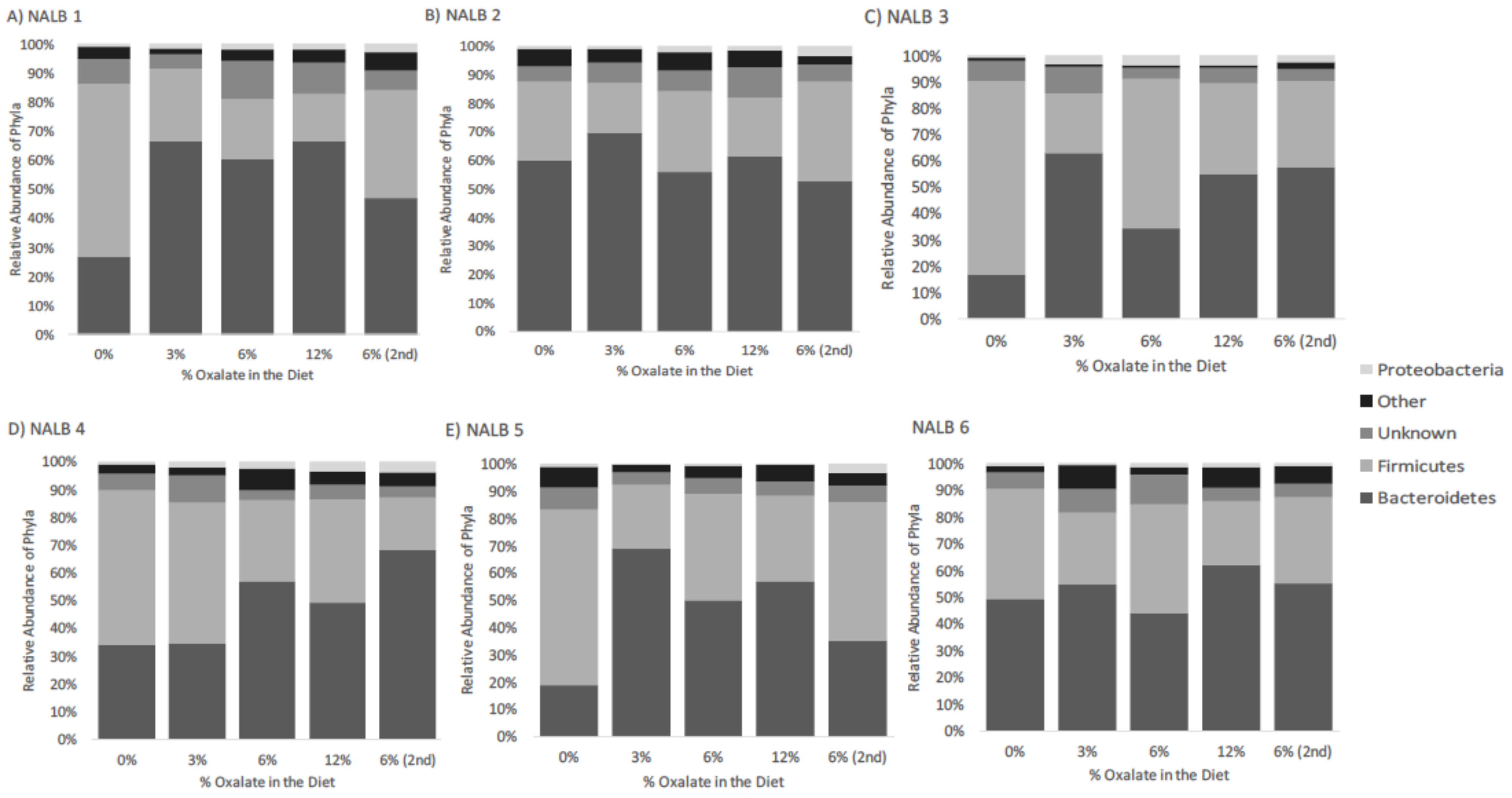

Figure S3. Phylum-level profile of the fecal microbiota over the duration of the experiment, for each animal that completed the trial. A) NALB 1; B) NALB 2; C) NALB 3; D) NALB 4; E) NALB 5; F) NALB 6.
