# Supplementary material for: Microbiota Diversification and Crash Induced by Dietary Oxalate in the Mammalian Herbivore Neotoma albigula
Source: mSphere. 2017 Oct 18;2(5):e00428-17. doi: 10.1128/mSphere.00428-17 (PMC5646245; doi:10.1128/mSphere.00428-17)
Supplement: TABLE S2 [file sph005172383st6.pdf]

Lowest assigned taxonomy	Taxonomic level	No. of OTUs	Relative abundance with diet of:		*rho*	*FDR*
			0% oxalate	12% oxalate		
Parabacteroides	genus	2	2.99 x 10^{5}	6.79 x 10^{5}	0.59 ± 0.001	0.015-0.016
Akkermansia	genus	2	1.92 x 10^{4}	2.78 x 10^{4}	0.56 ± 0.031	0.015-0.025
Bifidobacterium	genus	4	1.44 x 10^{4}	7.03 x 10^{4}	0.54 ± 0.04	0.012-0.045
Erysipelotrichaceae	family	1	3.01 x 10^{5}	3.91 x 10^{5}	0.53	0.026
Betaproteobacteria	class	4	4.22 x 10^{4}	1.8 x 10^{3}	0.52 ± 0.035	0.01-0.036
Coprococcus	genus	1	0	6.5 x 10^{6}	0.52	0.027
Oxalobacteraceae	family	2	5.53 x 10^{4}	2.31 x 10^{3}	0.51 ± 0.113	0.01-0.047
Allobaculum	genus	5	3.21 x 10^{5}	4.48 x 10^{4}	0.51 ± 0.012	0.025-0.038
Bacteria	kingdom	1	0	1.03 x 10^{5}	0.51	0.03
Alphapoteobacteria	genus	3	2.91 x 10^{4}	2.45 x 10^{3}	0.50 ± 0.02	0.025-0.036
Bacteroides	genus	6	6.62 x 10^{4}	2.19 x 10^{3}	0.46 ± 0.023	0.025-0.047
S24-7	family	336	4.6 x 10^{2}	1.35 x 10^{1}	0.46 ± 0.004	0.007-0.05
Unassigned	NA	436	9.73 x 10^{3}	2.8 x 10^{2}	0.46 ± 0.003	0.009-0.05
Marinilabiaceae	family	1	1.85 x 10^{5}	8.89 x 10^{5}	0.46	0.039
ML615J-28	order	6	3.99 x 10^{5}	2.23 x 10^{4}	0.45 ± 0.018	0.025-0.046
Ruminococcaceae	family	43	2.58 x 10^{3}	5.28 x 10^{3}	0.45 ± 0.009	0.01-0.05
Anaerostipes	genus	1	0	6.7 x 10^{6}	0.45	0.042
Bacteroidaceae	family	1	0	6.5 x 10^{6}	0.45	0.041
Lachnospiraceae	family	14	8.06 x 10^{6}	2.66 x 10^{4}	0.44 ± 0.016	0.012-0.05
RF39	order	11	1.33 x 10^{4}	1.76 x 10^{4}	0.44 ± 0.011	0.033-0.048
Ruminococcus (Ruminococcaceae)	genus	35	7.12 x 10^{3}	2.39 x 10^{2}	0.44 ± 0.008	0.017-0.05
YS2	order	5	1.64 x 10^{4}	2.2 x 10^{4}	0.43 ± 0.016	0.041-0.049
Rikenellaceae	family	7	1.86 x 10^{5}	5.74 x 10^{5}	0.43 ± 0.011	0.037-0.049
Oxalobacter	genus	1	0	3.61 x 10^{6}	0.43	0.045
Lactobacillus	genus	7	5.13 x 10^{4}	2.01 x 10^{3}	0.42 ± 0.007	0.044-0.049
Clostridiales	order	38	5.4 x 10^{4}	1.2 x 10^{3}	0.42 ± 0.005	0.027-0.05
RF16	family	7	6.06 x 10^{5}	7.25 x 10^{5}	0.42 +/- 0.008	0.038-0.048
Butyricimonas	genus	1	1.42 x 10^{5}	2.22 x 10^{5}	0.42	0.046
Christensenellaceae	family	1	0	8.69 x 10^{6}	0.42	0.046
Clostridium	genus	1	0	8.44 x 10^{6}	0.42	0.046
Mogibacteriaceae	family	1	0	7.34 x 10^{6}	0.42	0.46
Veillonella	genus	2	0	5.83 x 10^{5}	0.41 ± 0.013	0.045-0.047
Oscillospira	genus	9	6.1 x 10^{5}	1.36 x 10^{4}	0.41 ± 0.007	0.037-0.049
Haemophilus	genus	2	2.72 x 10^{5}	4.37 x 10^{5}	0.41 ± 0.003	0.046-0.047
Odoribacter	genus	1	1.71 x 10^{5}	2.36 x 10^{5}	0.41	0.047
RF32	order	2	2.79 x 10^{5}	3.67 x 10^{5}	0.40 ± 0.005	0.047-0.049
Desulfovibrio	genus	1	9.32 x 10^{6}	2.33 x 10^{5}	0.40	0.048
Ruminococcus (Lachnospiraceae)	genus	1	0	7.92 x 10^{5}	0.40	0.047
