# Supplementary material for: Microbiota Diversification and Crash Induced by Dietary Oxalate in the Mammalian Herbivore Neotoma albigula
Source: mSphere. 2017 Oct 18;2(5):e00428-17. doi: 10.1128/mSphere.00428-17 (PMC5646245; doi:10.1128/mSphere.00428-17)
Supplement: FIG S4 [file sph005172383sf4.pdf]

Figure S4.

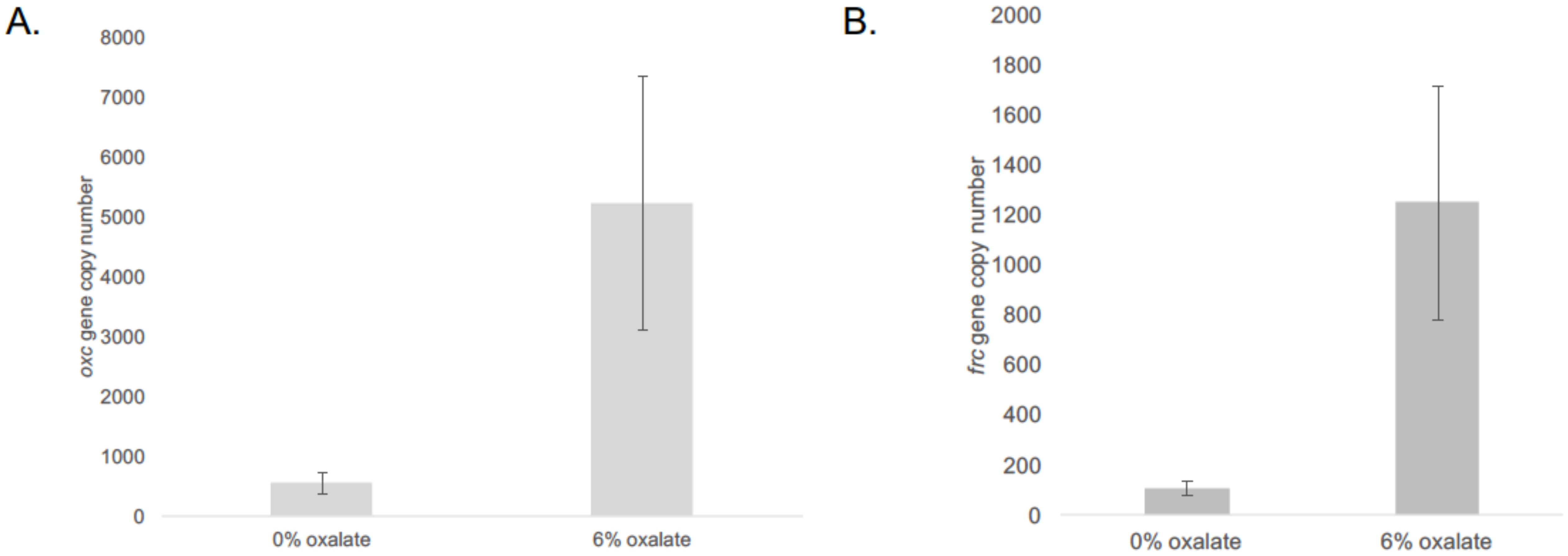

Figure S4. The abundance of the *oxc* (A) and *frc* (B) genes for the fecal microbiota after the 0% oxalate diet compared to after the first 6% oxalate diet. There was a significant increase in abundance for both the *oxc* ($t=2.675$, $df=5$, $p=0.047$) and *frc* ($t=2.879$, $df=5$, $p=0.031$) genes.
