# Supplementary material for: Microbiota Diversification and Crash Induced by Dietary Oxalate in the Mammalian Herbivore Neotoma albigula
Source: mSphere. 2017 Oct 18;2(5):e00428-17. doi: 10.1128/mSphere.00428-17 (PMC5646245; doi:10.1128/mSphere.00428-17)
Supplement: TABLE S3 [file sph005172383st7.pdf]

**# of OTUs**	**Taxon**	**Potential metabolic pathway**
1	*Clostridiium*	acetogenesis
36	*Ruminococcus*	acetogenesis
43	*Ruminococcaceae*	acetogenesis
9	*Oscillospira*	acetogenesis
14	*Lachnospiraceae*	acetogenesis
1	*Anaerostipes*	acetogenesis
1	*Coprococcus*	acetogenesis
1	*Lactococcus*	methanogenesis
1	*Odoribacter*	methanogenesis
1	*Desulfovibrio*	sulfate-reduction
